# Editorial: Immune-checkpoint inhibitors in anti-cancer armamentarium: a double-edged sword in risk of developing autoimmunity and immune-related adverse effects

**DOI:** 10.3389/fonc.2024.1476475

**Published:** 2024-08-26

**Authors:** Aysin Tulunay Virlan, Giacomo Cafaro, Flavia Sunzini, Yannick Degboe, Maria-Ioanna Christodoulou

**Affiliations:** ^1^ School of Infection & Immunity, University of Glasgow, Glasgow, United Kingdom; ^2^ Rheumatology Unit, Department of Medicine and Surgery, University of Perugia, Perugia, Italy; ^3^ Rheumatology Centre, Toulouse University Hospital and University Toulouse III, Toulouse, France; ^4^ Tumor Immunology and Biomarkers Laboratory, Basic and Translational Cancer Research Center, Department of Life Sciences, School of Sciences, European University Cyprus, Nicosia, Cyprus

**Keywords:** cancer immunotherapy, immune-checkpoint inhibitors (ICIs), immune-related adverse events (irAEs), autoimmunity, anti-CTLA-4, anti-PD-1/PD-L1

Immunotherapy represents a hopeful approach to managing cancer patients, mostly due to its relatively high safety and durable effects (1). Despite its benefits, emerged autoimmunity and immune-related adverse events (irAEs) are considered the vulnerable points of treatment with anti-CTLA-4 and/or anti-PD-1/PD-L1 antibodies (2). Additionally, individuals with pre-existing autoimmunity comprise a specific group of cancer patients who exhibit a high risk of autoimmune flare-up or irAEs upon IC inhibition (ICI) treatment (2). The incidence of autoimmune and/or immune-related complications is probably undervalued due to relatively short follow-ups, delayed onset, unclear etiology of symptoms, and cancer-associated death prior to their development (3). Furthermore, the landscape of cellular and molecular pathogenetic networks on the ground of immunotherapy is not fully understood. It is probably associated with series of events including expansion of low-avidity T cells and depletion of regulatory T cells, cross-presentation of neoantigens, and epitope spreading (2).

This Research Topic gathered up-to-date original articles, systematic reviews, reviews and case-reports on (auto)immune-mediated AEs in cancer patients upon mono- or combinatorial therapy with antibodies against ICs. Issues addressed regard their incidence, patient follow-up and monitoring, state-of-the art technologies for the elucidation of underlying pathogenetic mechanisms and detection of predictive biomarkers, as well as certain concerns about patients with pre-existing autoimmunity and misdiagnosis of irAEs.

The study by Li et al. provided for the first time data on ICI-associated toxicities in the hematologic and lymphatic systems. The authors worked on about 11.000 cases from more than 35 million reports deposited in the U.S. Food and Drug Administration Adverse Event Reporting System database and suggested that the incidence of hematologic and lymphatic AEs depends on the specific ICI drug, with anti-PD-1/anti-PD-L1 monotherapy linked with greater incidence compared to anti-CTLA-4 treatment, and atezolizumab (PD-L1 inhibitor) adding the highest risk. They also highlighted the involvement of sex dimension, reporting that these AEs appeared more often in female than male in ICI-treated patients. Anti-PD-1/PD-L1 therapy increases significantly the risk also for cardiovascular (CVD) AEs when administered with chemotherapy compared to chemotherapy alone, or as a monotherapy when compared to placebo, according to the systematic review by Zhang et al. Nevertheless, in this case dual therapy with anti-PD-1/PD-L1 plus anti-CTLA-4 antibodies correlates with a higher risk for CVD AEs than anti-PD-1/PD-L1 treatment alone.

The article by Lin et al. offered a comprehensive review of the current state of clinical research on immune checkpoint inhibition-related pneumonitis (CIP); a condition with ever growing incidence, not easily diagnosed due to individual-specific pathogenetic characteristics, and often related to poor prognosis. The authors summarized the risk factors associated with CIP, which include underlying lung disease and smoking that associate with a pro-inflammatory status in the lung, the use of anti-PD-1 rather than anti-PD-L1 regimens, prior radiotherapy associated with DNA damage and secretion of pro-inflammatory cytokines and chemokines, pre-existing autoimmunity or infection, and the type of cancer e.g. squamous cell carcinoma. Immunophenotyping of the inflamed lung tissue, peripheral blood and the bronchoalveolar lavage fluid provide useful predictive and diagnostic indicators of CIP in patients with solid tumors treated with ICI. The authors also reported current advances in the CIP management including pulsed corticosteroid therapy, or, in the cases of steroid-refractory CIP, tocilizumab (anti-human IL-6 receptor antibody) or nintedanib (anti-VEGF/anti-FGFR/anti-PDGFR small molecule tyrosine-kinase inhibitor). However, all the above strategies are in need of further exploration.

In the context of predictive biomarkers, Cao et al., listed a nice summary of cutaneous irAEs-specific indicators per tumor type, that can be assessed in the serum, peripheral blood, or the tumor site of patients. Captivatingly, the authors highlighted the role of multi-omics approaches in the elucidation of pathogenetic mechanisms and pinpointing key deregulated molecular networks in cutaneous irAEs, and further promoting the discovery of potential predictive biomarkers towards precise and personalized clinical approaches. Indicatively, they reviewed proposed single- or multi-gene and protein models, immunophenotyping panels, HLA haplotypes, miRNA/SNPs and tumor microenvironment (TME) parameters for the prediction of ICI-induced AEs or resistance to ICIs, all unraveled upon genomics, transcriptomics, proteomics, and radiomics research. Importantly though, the authors mentioned the need for future confirmation of the predictive ability of the above in larger clinical cohorts and the development of laboratory assays that can be used in clinical routine practice.

Another aspect of the ICI-induced irAEs was the focus of the systematic review article by Liang et al., who explored their effect on survival rates of patients with non-small cell lung cancer. Remarkably, the study revealed that mild and early irAEs (but not severe ones) associate with better overall (OS) and progression-free survival (PFS) compared to the absence of them. Also, irAEs affecting the skin and the endocrine system tend to associate with more favorable survival prognosis than hepatitis or those affecting the gastrointestinal tract or the lung.

Three case reports were also included in this Research Topic. In the first, Daetwyler et al. described the case of a male patient with Hodgkin’s lymphoma who after the 6^th^ cycle of treatment with pembrolizumab (anti-PD-1 antibody), developed monocular optic neuropathy, treated successfully with high-dose steroid therapy. Yet, when the second eye was affected, amelioration of symptoms was managed after extended combinatory immunosuppressive therapy. Xiong et al. described the cases of two cancer patients with pre-existing paraneoplastic dermatomyositis and “seronegative” paraneoplastic demyelinating neuropathy, respectively, treated with ICIs, the effectiveness of which were not compromised by intravenous immunoglobulin used for the neuromuscular events. On the occasion of these cases, the authors conducted also a review of the related literature. Lastly, Torres-Zurita et al. reported the case of a patient with advanced, metastatic hepatocellular carcinoma, undergoing second-line treatment with nivolumab (anti-PD-1) and ipilimumab (anti-CTLA-4 antibody), who developed sarcoidosis-like reaction (SLR). The authors emphasized the fact that the SLR mimics disease progression, and raised awareness about possible misdiagnosis.

In summary, this Research Topic gathered articles on the current biomedical and clinical research on irAEs being developed in ICIs-treated cancer patients, and adds significant value to the existing knowledge about the utmost importance for their optimal prediction, diagnosis and management, given that these therapeutic approaches become ever more widely used.

## References

[1] Hoos A. Development of immuno-oncology drugs - from CTLA4 to PD1 to the next generations. Nat Rev Drug Discovery. (2016) 15:235–47. doi: 10.1038/nrd.2015.35 26965203

[2] June CH Warshauer JT Bluestone JA. Is autoimmunity the Achilles’ heel of cancer immunotherapy? Nat Med. (2017) 23:540–7. doi: 10.1038/nm.4321 28475571

[3] Freeman-Keller M Kim Y Cronin H Richards A Gibney G Weber JS. Nivolumab in resected and unresectable metastatic melanoma: characteristics of immune-related adverse events and association with outcomes. Clin Cancer Res. (2016) 22:886–94. doi: 10.1158/1078-0432.CCR-15-1136 PMC4755809 26446948

